# Individualized penetrating keratoplasty using edge-trimmed glycerol-preserved donor corneas for perforated corneal ulcers

**DOI:** 10.1186/s12886-019-1091-4

**Published:** 2019-04-02

**Authors:** Guozhen Niu, Qi Zhou, Xinyu Huang, Sangsang Wang, Juan Zhang, Yushan Zhang, Yanlong Bi

**Affiliations:** Department of Ophthalmology, Tongji Hospital, Tongji University School of Medicine, 389 Xincun Road, Shanghai, 200065 China

**Keywords:** Individualized penetrating keratoplasty, Preserved, Cornea, Corneal perforation

## Abstract

**Purpose:**

To report a surgical technique and the surgical outcomes of individualized penetrating keratoplasty (PK) using edge-trimmed glycerol-preserved donor corneas for perforated corneal ulcers.

**Methods:**

Fourteen perforated eyes from 14 patients who underwent individualized PK using edge-trimmed glycerol-preserved donor corneas, were included in the retrospective study. The perforations were mainly 1–2 mm in size except for one that was 2.5 × 4 mm. Three patients were treated with PK; one patient was treated with PK and a conjunctival flap; ten patients who had large ulcer areas were treated with PK combined with lamellar keratoplasty (LK). Donor corneas were preserved in sterile pure glycerol at − 80 °C. Corneal grafts were specially edge-trimmed to match the perforation, and then sutured onto the recipient bed avoiding the visual axis.

**Results:**

All 14 patients recovered anatomical integrity without reinfections of the treated eyes. All patients had improved graft transparency and uncorrected visual acuity after surgery. Among them, four patients suffered from short-term postoperative complications and recovered quickly; four patients suffered from long-term postoperative complications, of them, one was performed further treatment.

**Conclusion:**

After individualized PK using glycerol-preserved donor corneas, all perforated corneal ulcers were stably controlled by the end of the follow-up period. This modified surgical technique can be a potential treatment choice for patients with perforated corneal ulcers.

## Background

Corneal perforation is the destruction of the globe integrity of the cornea, and can lead to profound vision loss and severe ocular morbidity [1–3]. When corneal perforation occurs, patients need appropriate and immediate treatments to preserve the anatomic integrity of the cornea, and to prevent complications such as secondary glaucoma or endophthalmitis [3]. Many treatments can be used in perforated corneal disorders, including nonsurgical approaches (limiting inflammation, treating a coexistent infection, anti-collagenase and antiglaucoma treatment, optimizing epithelial healing using bandage soft contact lenses and autologous serum eye drops [3–5], and pressure bandaging), and surgical treatments (tissue adhesives, amniotic membrane transplants (AMT), conjunctival flaps, pericardial membranes, tarsorrhaphy, therapeutic ptosis with botulinum toxin, lamellar keratoplasty (LK), and penetrating keratoplasty (PK) [3, 4, 6–12]). The treatment selection mainly depends on the size and location of the perforation and the status of the underlying disease [3]. One of the most common selection is PK [13].

PK requires viable donor corneas [12, 14–19] to replace full-thickness corneas [20]. However, the shortage of fresh donor corneas is still a nonnegligible issue in many countries [13, 21]. And sometimes the corneal perforation is too urgent to withstand the wait for fresh corneas. Researchers have found that glycerol can preserve acellular corneal tissues simply and effectively for up to 5 years [21]. These acellular glycerol-preserved corneas may be used in PK when fresh donor corneas are not available [22]. In addition, some researchers have found that the residual corneal endothelial cells beyond the perforation may migrate and cover the posterior graft surface after PK with glycerol-preserved corneas [13]. Therefore, an increasing number of investigators in different countries are trying to use glycerol-preserved donor corneas for therapeutic penetrating keratoplasty (TPK) in patients with corneal perforations [13, 22, 23].

This study presents individualized PK using edge-trimmed glycerol-preserved donor corneas for perforated corneal ulcers. This technique can be a choice to treat corneal ulcers with perforations.

## Methods

### Patients and donor corneas

A retrospective study was performed by reviewing medical records from the Department of Ophthalmology, Tongji Hospital, Tongji University School of Medicine. Fourteen patients, who suffered from perforated corneal ulcers and were treated with individualized PK using edge-trimmed glycerol-preserved donor corneas from September 2015 to August 2017, were included. All patients had relatively normal function of corneal endothelium, no hypopyon, and no history of cataract surgery. Patients with fulminant corneal infections and rapidly progressing perforations such as *Pseudomonas aeruginosa* infection, and with a wide range of cornea melting such as fungal keratitis, were excluded.

**Fig. 1:**
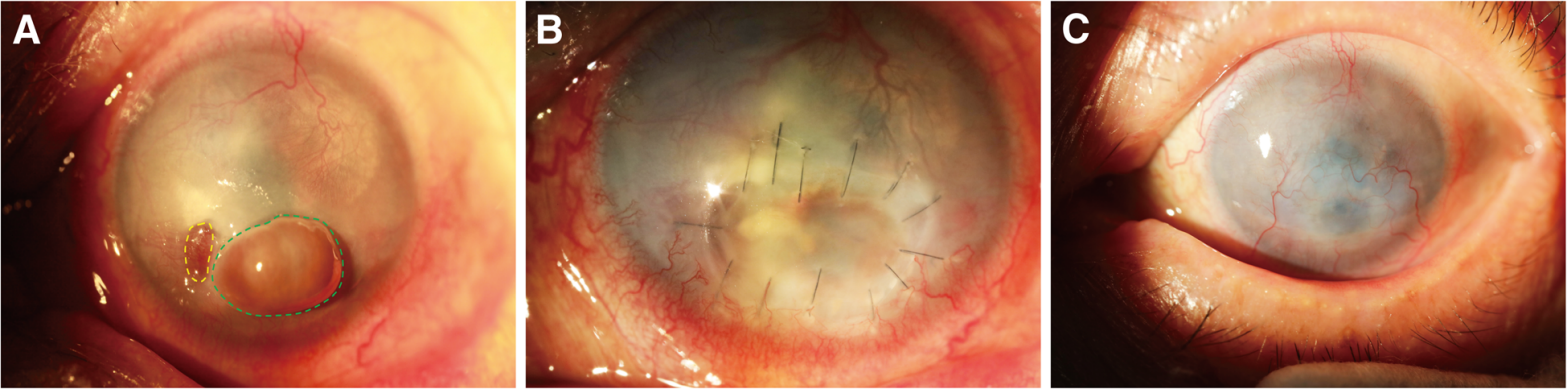
Patient 5 (necrotizing stromal keratitis (HSK), visual acuity: HM, left eye). **a** Green dotted circle indicates the perforation (4 × 2.5 mm in diameter) with iris prolapse; yellow dotted circle indicates a small corneal ulcer beside the perforation. **b** An edge-trimmed PK graft covered both perforated and ulcerated regions was sutured onto the recipient bed. **c** 16 months after surgery, the graft was stable, with improved transparency

**Fig. 2:**
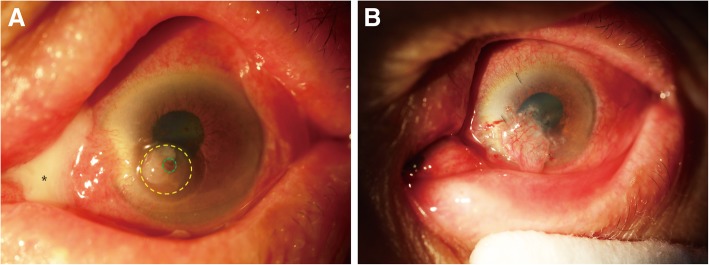
Patient 1 (upper lacrimal duct inflammation, ophthalmodynia and massive secretions, left eye). **a** Green dotted circle indicates the perforation (1 × 1 mm in diameter) with iris prolapse; yellow dotted circle indicates a corneal ulcer; asterisk indicates a large amount of white purulent secretion emitted from the upper lacrimal punctum. **b** After edge-trimmed PK surgery, a conjunctival flap was used to cover the corneal graft to avoid corneal reinfection

The study included seven male and seven female patients, with an average age of 56.93 ± 10.94 years (age range: 42–85 years). Four patients had infectious corneal ulcers, and the other ten patients had noninfectious corneal ulcers (autoimmune causes). The corneal perforations in these patients were mainly 1–2 mm in size except for one large perforation with a size of 2.5 × 4 mm (Fig. 1a). Thirteen perforations were eccentric of the cornea, and one was centric (Table 1). Three patients were treated with PK (Fig. 1); one patient was treated with PK and a conjunctival flap (Fig. 2); ten patients with large ulcer areas were treated with PK combined with LK (Figs. 4 and 5). Corneal grafts were trimmed into wedged edged shape—usually 0.25–0.5 mm larger in diameter [13]—according to the base size of the corneal perforations, and then sutured watertight onto the recipient bed, avoiding the visual axis.

**Table 1:** Summary of Clinical Data and Graft Information

Patient Number	Age and Sex	Onset Time	Cause	Perforation Number and Size (mm^2^)	Donor Size of Penetrating Corneal Graft (mm^2^)	Location	Procedure
1	85/ M	2 weeks	Infectious (Bacteria)	1/ 1 × 1	1.5 × 1.5	Eccentric	PK + conjunctival flap
2	63/ M	3 weeks	Infectious (Bacteria)	1/ 2 × 2	2.5 × 2.5	Eccentric	PK
3	47/ F	40 years	Noninfectious (No factors)	1/ 1.5 × 1.5	2 × 2	Eccentric	PK + LK
4	42/ F	2 months	Noninfectious (Autoimmune)	1/ 1.5 × 1.5	2 × 2	Centric	PK + LK
5	64/ M	1 month	Infectious (Virus)	1/ 4 × 2.5	4.5 × 3	Eccentric	PK
6	48/ F	2 years	Noninfectious (Autoimmune)	1/ 2 × 2	2.5 × 2.5	Eccentric	PK + LK
7	62/ F	4 days	Noninfectious (Autoimmune)	2/ 1 × 1	1.5 × 1.5	Eccentric	PK + LK
8	53/ F	3 weeks	Noninfectious (Autoimmune)	1/ 1 × 1	1.5 × 1.5	Eccentric	PK + LK
9	58/ F	5 days	Noninfectious (Autoimmune)	1/ 1.5 × 1.5	2 × 2	Eccentric	PK + LK
10	49/ M	3 months	Noninfectious (Autoimmune)	1/ 1 × 1	1.5 × 1.5	Eccentric	PK + LK
11	67/ M	1.5 months	Noninfectious (Autoimmune)	1/ 2 × 2	2.5 × 2.5	Eccentric	PK + LK
12	52/ M	1 week	Infectious (Bacteria)	1/ 1 × 1	1.5 × 1.5	Eccentric	PK
13	50/ M	10 months	Noninfectious (Autoimmune)	1/ 1.5 × 1.5	2 × 2	Eccentric	PK + LK
14	57/ F	2 weeks	Noninfectious (Autoimmune)	1/ 2 × 2	2.5 × 2.5	Eccentric	PK + LK

*M* male, *F* female, *PK* penetrating keratoplasty, *LK* lamellar keratoplasty

The donor corneas were preserved in sterile pure glycerol at − 80 °C for 6–12 months.

### Surgical process

**Fig. 3:**
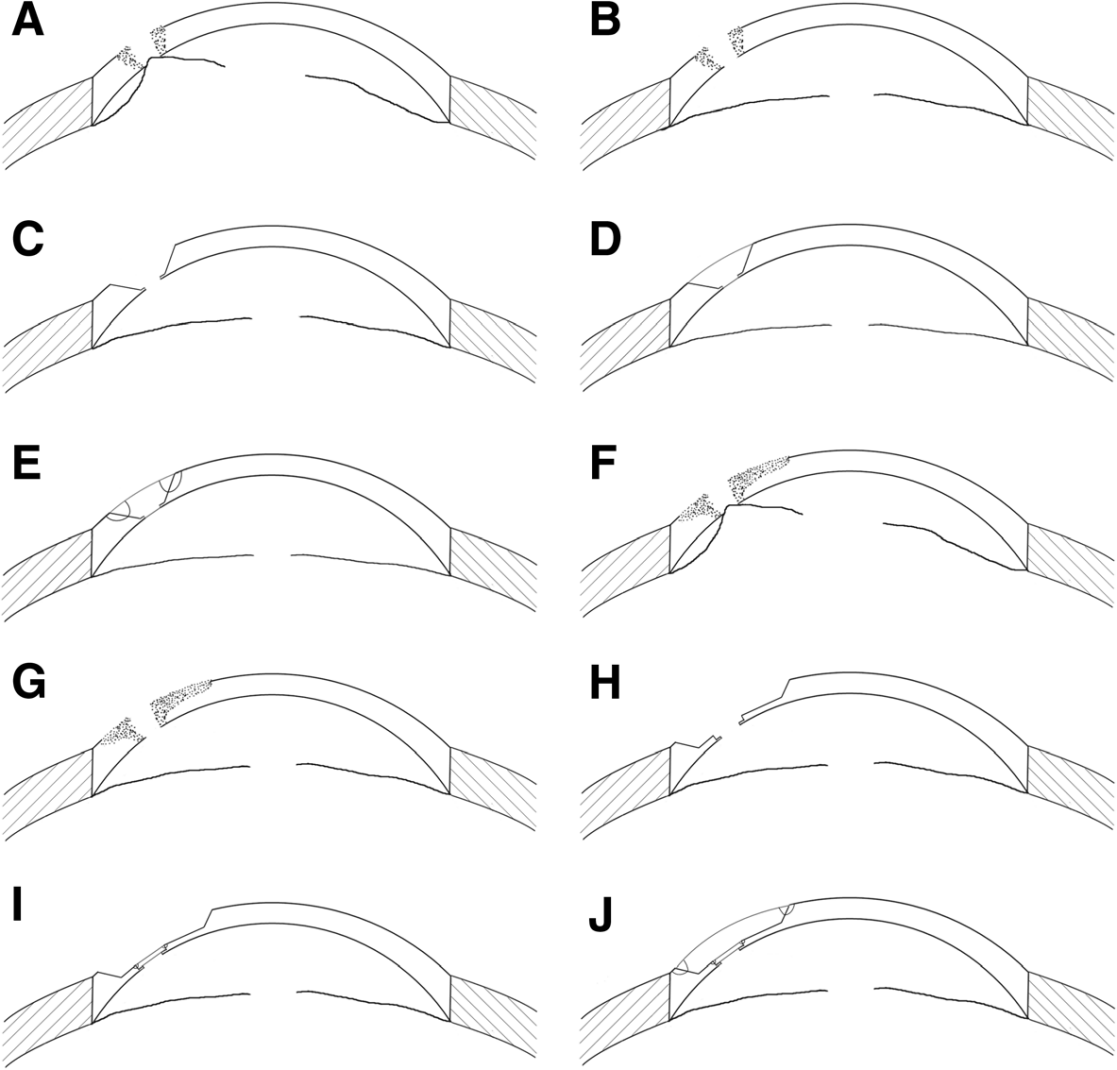
Schematic diagrams for surgical steps. **a** Perforation with iris prolapse and shallow anterior chamber. **b** Iris and anterior chamber restored after Healon injection. **c** The recipient bed was produced with sloping surface by excising the necrotic tissue and trimming the perforation edge. **d** Trimmed corneal graft with wedged edge was inserted into the recipient bed. **e** Watertight suture. **f** Perforation with iris prolapse, shallow anterior chamber and large corneal ulcer. **g** Iris and anterior chamber restored after Healon injection. **h** The recipient bed was produced by excising the necrotic tissue and trimming the perforation edge. **i** A thin PK graft (approximately 100 μm) was transplanted to the perforated area with watertight suture. **j** A thicker and larger-edged trimmed LK graft was sutured onto the region of the corneal ulcer

Conjunctival anesthesia was performed using 0.5% proparacaine hydrochloride—topically instilling 4–5 drops into conjunctival sac [24]. Retrobulbar anesthesia was performed using a mixture of 2% lidocaine (2 ml) and 0.75% bupivacaine (2 ml) without pressing the eye. All procedures were performed gently. All patients had iris prolapses and shallow anterior chambers. To restore these symptoms, we made a 1.0 mm limbal stab incision using a 15-degree tipped blade, and then injected miotic solution and sodium hyaluronate (10 mg/mL Healon) into the anterior chamber through the incision [13] (Fig. 3a–b, f–g).

For cases that ulcer areas around the perforation was small, we carefully excised necrotic tissue around perforation and ulcer to build a recipient bed with sloping surface (Fig. 3c). Then we marked a matched outline on the posterior surface of donor cornea using corneal trephines (usually 0.25–0.5 mm larger [13]), and trimmed the graft into wedged edge and proper thickness and shape along the mark using an ophthalmic surgical blade (#11) to match the recipient bed. At last, the graft was sutured onto the bed using interrupted suture with 10–0 nylon suture, avoiding the visual axis, and was checked for water tightness (Fig. 3d–e).

For cases that ulcer areas around the perforation was large, as shown in Fig. 3f, we treated the patients with PK combined with LK. After the necrotic tissue was excised (Fig. 3h), we sutured a thin penetrating corneal graft (approximately 100 μm in thickness) to the perforated area as inner layer (Fig. 3i), then we trimmed to get a lamellar graft according to the shape of the ulcerated region, and patched it onto the recipient bed (with a sloping surface) as outer layer, and fixed it using interrupted suture with 10–0 nylon suture (Fig. 3j).

### Medications

Preoperatively, patients with infectious perforated corneal ulcers were given systemic and topical antimicrobial treatments directed against pathogens (including cefathiamidine injection, 0.5% levofloxacin eye drops, 0.3% tobramycin eye drops, acyclovir tablets, 0.1% acyclovir eye drops and 5 g/ 7.5 mg ganciclovir ophthalmic gel). Patients with autoimmune corneal ulcers were routinely given broad-spectrum antibiotic therapy (including cefathiamidine injection and 0.5% levofloxacin eye drops). Thirty minutes before surgery, patients were commonly given 250 ml 20% mannitol through intravenous drip.

Postoperatively, patients were given systemic and topical broad-spectrum antibiotic therapies (including cefathiamidine injection and 0.5% levofloxacin eye drops), and other antimicrobial treatments according to the pathogens (including acyclovir tablets, 0.1% acyclovir eye drops and 5 g/ 7.5 mg ganciclovir ophthalmic gel). In addition, they were treated with systemic and topical steroids and topical immunosuppressive eye drops (1% cyclosporine eye drops) when necessary.

## Results

Clinical courses are summarized in Table 2. All patients were followed up from the first visit to at least corneal stabilization after surgery (13.86 ± 3.01 months; 9–20 months). The mean re-epithelization time was 4.15 days. All PK grafts (all patients) became transparent without edema with a mean time of 3.96 months, whereas LK grafts (patients received PK combined with LK) became transparent without edema with a mean time of 0.55 months.

**Table 2:** Summary of the Clinical Courses

Patient Number	Preoperative UCVA	Postoperative UCVA	Re-epithelialization Time (d)	Transparency Recovery Time (mo)		Complications and Treatments		Follow-up Time (mo)
				LK graft	PK graft	Short-term	Long-term	
1	HM/ 10 cm	HM/ 20 cm	–	–	3	High IOP (2 days after surgery)/Anterior chamber irrigation	–	9
2	LP	CF/ 30 cm	7	–	4	High IOP (2 days after surgery)/ Topical antiglaucoma medications	Aggravation of Lens Opacification (3 months after surgery)/ No treatment	13
3	LP	0.06	5	0.5	3.5	Double anterior chamber (2 days after surgery)/ Strengthen the PK graft with more sutures	–	15
4	HM/ 20 cm	0.1	3	0.25	5	Double anterior chamber (2 days after surgery)/ Recovery with no treatment	–	15
5	HM/ 10 cm	HM/ 40 cm	4	–	6	–	Aggravation of Lens Opacification (4 months after surgery)/ No treatment	16
6	CF/ 30 cm	0.08	4	0.5	4	–	Aggravation of Lens Opacification (3 months after surgery)/ No treatment	14
7	CF/ 30 cm	0.25	6	1	4	–	Micro-perforation (1.5 months after surgery)/ Conjunctival flap transplantation	13
8	LP	0.12	2	0.5	3	–	–	14
9	CF/ 40 cm	0.1	4	0.75	4	–	–	16
10	CF/ 30 cm	0.15	3	0.5	3.5	–	–	12
11	HM/ 10 cm	CF/ 30 cm	5	0.75	4.5	–	–	10
12	LP	0.04	4	–	3	–	–	20
13	HM/ 30 cm	0.15	3	0.25	3	–	–	10
14	LP	0.04	4	0.5	5	–	–	17

*HM* hand motion, *CF* counting finger, *LP* light perception, *UCVA* uncorrected visual acuity, *IOP* intraocular pressure

**Fig. 4:**
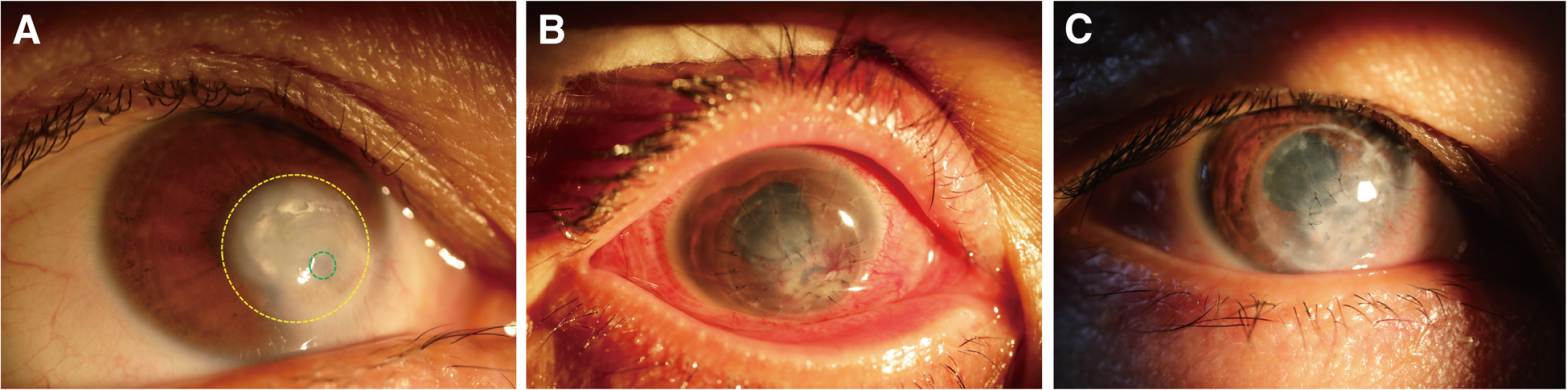
Patient 3 (noninfectious, visual acuity: LP, right eye). **a** Green dotted circle indicates the perforation (1.5 × 1.5 mm in diameter) with a small area of iris prolapse; yellow dotted circle indicates a large circular area of corneal ulcer and leucoma. **b** A small, thin PK graft was sutured to close the perforation, then a LK graft—larger than the ulcerated region—was sutured across the optical axis for a better visual acuity after surgery. **c** 9 months after surgery, the graft showed improved transparency

**Fig. 5:**
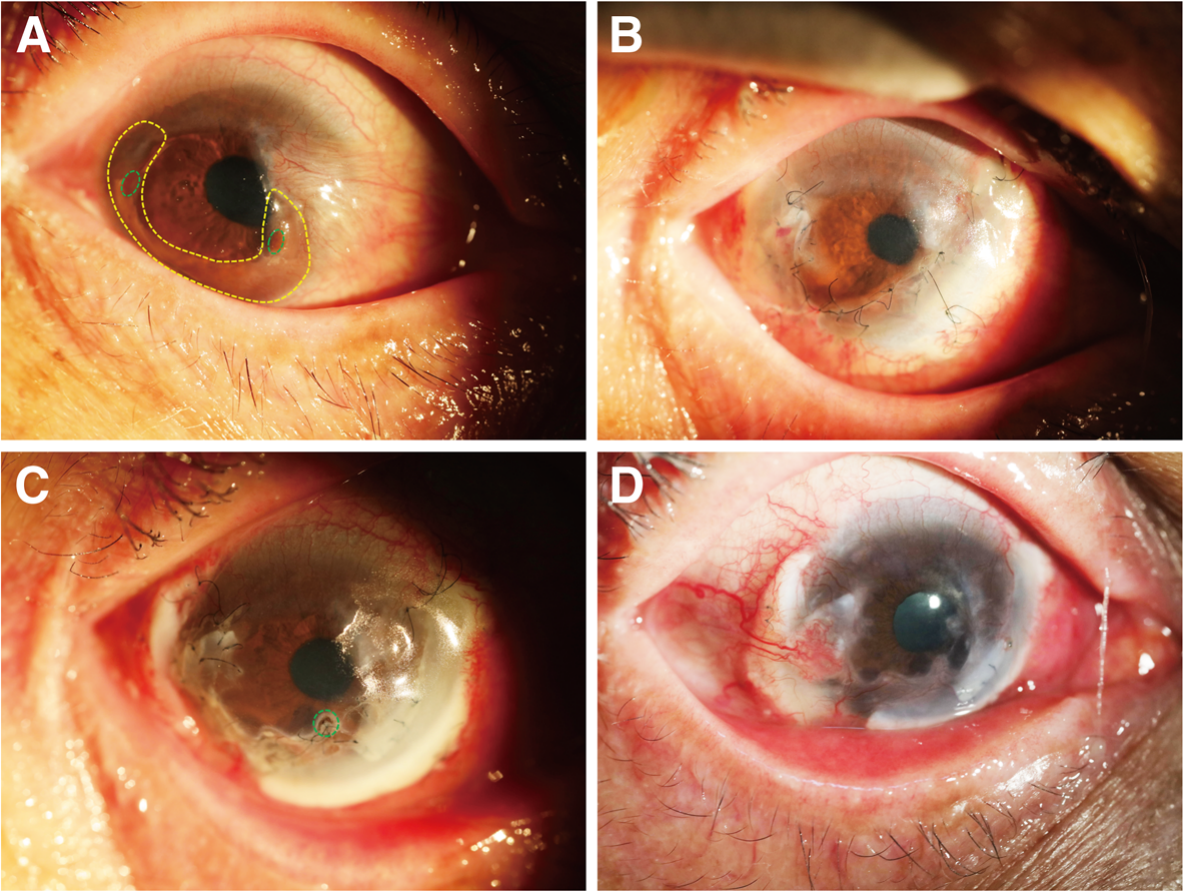
Patient 7 (Mooren’s ulcer, visual acuity: CF, right eye). **a** Green dotted circle indicates two perforations (1 × 1 mm in diameter) with prolapsed iris; yellow dotted circle indicates a large crescent ulcer at the peripheral region; a pterygium was at the nasal side. **b** The pterygium was excised, and individualized PK was performed with two thin, penetrating corneal grafts to cover the perforations. A crescent LK graft covered the PK grafts and the ulcerated areas around the perforations, including the region where pterygium was excised. **c** Green dotted circle indicates a micro-perforation (1.5 months after the surgery), which was cured by the transplantation of a conjunctival flap. **d** After a follow-up period of 13 months, the graft showed improved transparency

After surgery, four patients (Patient 1–4; 28.57%) suffered from short-term complications. Patient 1 and Patient 2 suffered from high intraocular pressure (IOP) 2 days after surgery, Patient 1 was treated with anterior chamber irrigation and Patient 2 was treated by topical application of antiglaucoma medications. Patient 3 (Fig. 4) and Patient 4 experienced double anterior chambers 2 days after surgery. The complication of Patient 3 was solved by strengthening the inner PK graft with more sutures. The complication of Patient 4 was resolved without treatment. After surgery, four patients (Patients 2, 5, 6 and 7; 28.57%) suffered from long-term complications. Patient 2, Patient 5 and Patient 6 experienced aggravation of lens opacification with a mean time of 3.3 months after surgery. In Patient 7, a micro-perforation occurred 1.5 months after surgery (Fig. 5c), and was cured after the transplantation of a conjunctival flap.

At the end of the follow-up period, all patients recovered anatomical globe integrity without reinfections of the treated eyes. All patients had improved postoperative uncorrected visual acuity (UCVA) after surgery, ranging from HM/ 20 cm to 0.25. All patients also had improved corneal graft transparency without corneal edema after surgery.

## Discussion

In this study, the modified surgical technique was successfully performed in 14 patients. All patients recovered anatomical globe integrity postoperatively. By the end of follow-up, treatments with these glycerol-preserved corneal grafts cured the perforated ulcers with improved UCVA, avoiding a worse result of globes evisceration. We performed individualized PK using edge-trimmed glycerol-preserved donor corneas for perforated corneal ulcers. This technique can be a choice to treat corneal ulcers with perforations.

Glycerol is a dehydrating agent with antimicrobial and antiprotease properties. However, endothelial viability—a condition needed for optical PK—is not preserved in glycerol [21]. Compared with fresh donor corneas, glycerol-preserved corneas have several potential advantages, such as increased availability, weak graft rejection and low risk of microbial contamination [21]. Glycerol-preserved corneas, with a graft size ranging from 2 to 8 mm [22] and 7 to 10 mm [23], had been applied for TPK in infectious keratitis, which showed postoperative complications such as reinfection, secondary PK, opacification or glaucoma. Shi et al. [13] reported cases treated eccentric corneal perforations by PK using glycerol-preserved small-diameter corneal grafts (2.5–4.5 mm), which showed all grafts remained clear at the end of a follow-up period from 7 to 36 months. The above studies suggested that glycerol-preserved donor corneas can be used as emergency transplants when fresh donor corneas are not available. For safety considerations, we excluded patients with fulminant corneal infections, rapidly progressing perforations and large areal cornea melting. For better postoperative visual function and avoidance of residual corneal endothelial decompensation, we had just tried corneal grafts ranged from 1.5 to 4.5 mm for keratoplasty. We believe that this technique also has advantages in treating patients with large perforation areas and some severe cases. To further evaluate the safety and prognosis of the procedure, more efforts are needed.

For patients with small ulcer areas of corneal perforation, the one-layer, wedged-edge, trimmed PK graft was enough to fit the recipient bed and helpful for corneal re-epithelialization and re-endothelialization. For patients with large corneal ulcer areas around small perforations, the one-layer PK procedure could not only lead to remove more corneal endothelial cells, but also lead to an extensive and continuous edema between the corneal graft and the recipient bed, potentially causing further decompensation of the recipient corneal endothelium and transplant failure. The technique combining PK with LK could override these disadvantages in the situation.

Although this technique is slightly complicated, it has potential advantages. The trimmed edge of the graft enlarged the contact area between the donor and recipient, increased the stability of the corneal graft and reduced the leakage of aqueous humor through the suture. The trimmed graft edge also created large front surface on the corneal graft, which would promote graft re-epithelization and reduce corneal astigmatism after surgery.

In our study, we mainly evaluated the corneal endothelial function by measuring central corneal thickness (using AS-OCT) and observing the transparency of cornea and the graft. After surgery, the residual corneal endothelial cells may migrate to cover the posterior surface of the grafts [13]. Corneal endothelial cell counting before and after the procedure by in vivo Confocal Microscopy could have be done to better evaluate the corneal endothelial function and the prognosis of the procedure. As a retrospective study, unfortunately, the clinical indexes (including the corneal endothelial cell count before and after surgery, the AS-OCT results) were not fully obtained. During the preparation of the recipient bed, we excised necrotic tissue with the least loss of endothelial cells, which could contribute to the improvement of postoperative visual function. The perforation size is also negative correlated with the number of residual recipient corneal endothelial cells. Thus, it is very meaningful to explore the relationship between the recipient’s endothelial cell number and the postoperative visual function.

However, there were some limitations in the study. In PK combined with LK, the sutures fixing the LK graft will be removed after the stabilization of the corneal graft, whereas the deeper sutures fixing the PK graft will remain. If the inner sutures are centric, they may affect visual acuity after surgery. As a retrospective study with a relatively small sample size, the possible selective bias was inevitable. Control group should be included in future studies to make our results more convincing. In addition, the current study would have benefitted from a longer follow-up time. Further investigations will help to improve the potential effectiveness and benefits of this surgical technique.

## Conclusion

In summary, individualized PK using edge-trimmed glycerol-preserved donor corneas can successfully treat perforated corneal ulcers. The technique can be a treatment choice for patients with perforated corneal ulcers, especially in cases with large area of corneal ulcers around the perforations and in lack of fresh donor corneas.

## Abbreviations

AMT: Amniotic membrane transplants
CF: Counting finger
HM: Hand motion
IOP: Intraocular pressure
LK: Lamellar keratoplasty
LP: Light perception
PK: Penetrating keratoplasty
TPK: Therapeutic penetrating keratoplasty
UCVA: Uncorrected visual acuity
